# LncRNA CACNA1G-AS1 up-regulates FTH1 to inhibit ferroptosis and promote malignant phenotypes in ovarian cancer cells

**DOI:** 10.32604/or.2023.027815

**Published:** 2023-04-10

**Authors:** YANPING JIN, JIANPING QIU, XIUFANG LU, YAN MA, GUOWEI LI

**Affiliations:** 1 Department of Obstetrics and Gynecology, The Affiliated Suzhou Hospital of Nanjing Medical University, Suzhou Municipal Hospital North, Suzhou, China; 2 Department of Neurosurgery, The Second Affiliated Hospital of Soochow University, Suzhou, China

**Keywords:** Ovarian cancer, m6A methylation, Ferroptosis, Mitophagy, Malignant phenotype

## Abstract

Previous study revealed that ferritin heavy chain-1 (FTH1) could regulate ferritinophagy and affect intracellular Fe^2+^ content in various tumors, while its N6-methyladenosine (m6A) RNA methylation was closely related the prognosis of ovarian cancer patients. However, little is known about the role of FTH1 m6A methylation in ovarian cancer (OC) and its possible action mechanisms. In this study we constructed FTH1 m6A methylation regulatory pathway (LncRNA CACNA1G-AS1/IGF2BP1) according to related bioinformatics analysis and research, through clinical sample detections we found that these pathway regulatory factors were significantly up-regulated in ovarian cancer tissues, and their expression levels were closely related to the malignant phenotype of ovarian cancer. *In vitro* cell experiments showed that LncRNA CACNA1G-AS1 could up-regulate FTH1 expression through IGF2BP1 axis, thus inhibited ferroptosis by regulating ferritinophagy, and finally promoted proliferation and migration in ovarian cancer cells. Tumor-bearing mice studies showed that the knock-down of LncRNA CACNA1G-AS1 could inhibited the tumorigenesis of ovarian cancer cells *in vivo* condition. Our results demonstrated that LncRNA CACNA1G-AS1 could promote the malignant phenotypes of ovarian cancer cells through FTH1-IGF2BP1 regulated ferroptosis.

## Introduction

Until now, the clinical treatment of this ovarian cancer has not achieved satisfactory effects; after initial treatment, approximately 70%–80% of patients suffer tumor recurrence [1–3]. Thus, the identification of new targets for ovarian cancer is crucial to prevent tumor progression and improve curative effects in these cases.

Iron overload can lead to excessive Fe^2+^ ions participating in the Fenton reaction to produce hydroxyl radicals, and if there is an intracellular antioxidant deficiency, hydroxyl radicals will be high, leading to oxidative stress reactions, which can produce a large number of reactive oxygen species (ROS) and trigger ferroptosis. Previous research revealed that abnormal intracellular iron content was closely related to the occurrence of various tumors (including lung, liver and ovarian cancer), which suggested that interfering with iron metabolism during the early stages of ovarian cancer may be an effective way to kill tumor cells. Moreover, relevant studies have revealed that the expression of lipid ROS in ovarian cancer cells is significantly dysregulated compared to that in normal cells [4–7]. Therefore, some scholars believe that ferroptosis may play a key role in ovarian cancer progression.

Ferritin can store Fe^2+^ in cells and can be transported to lysosomes via autophagy; it binds to nuclear receptor coactivator 4 (NCOA4) to facilitate its degradation and the release of free Fe^2+^ by ferritin heavy chain 1 (FTH1); this process is called ferritinophagy. Scholars have found that ferritinophagy can regulate intracellular Fe content and participate in the excessive accumulation of ROS induced by the Fenton reaction and then take part in pathological processes such as proliferation, differentiation and programmed cell death by mediating ferroptosis. Bioinformatics analysis showed that FTH1 shared high sensitivity and accuracy in predicting the prognosis of ovarian cancer patients [8–11]. Therefore, we speculated that FTH1 could regulate ferroptosis through the ferritinophagy axis, thus affecting the malignant phenotype of ovarian cancer.

Previous bioinformatics analysis showed that the expression levels of FTH1 methylation and m6A methylation regulators were obviously correlated with the prognosis of ovarian cancer patients. IGF2 mRNA binding protein 1 (IGF2BP1) belongs to the IGF binding protein family, and an ovarian cancer risk score model showed that IGF2BP1 shared great value in the prognostic analysis of ovarian cancer cases. Patients with high IGF2BP1 expression exhibited a lower survival rate and worse clinical prognosis than those with low expression [12]. LncRNA CACNA1G-AS1 can target IGF2BP1, thus regulating the expression of target mRNAs. Zheng confirmed that CACNA1G-AS1 is strongly linked to the methylation profile and clinical prognosis of ovarian cancer patients through bioinformatics analysis [13]. Based on these results, in our study, we attempted to construct an FTH1 methylation regulatory network and explore its mechanism of action in ferroptosis and the malignant phenotypes of ovarian cancer at the molecular level by experimental analysis.

## Materials and Methods

### Cell culture

Ovarian cancer cells (SKOV3 and A2780) were provided by the Shanghai Institute of Chinese Academy of Sciences. All cells were cultured in RPMI-1640 medium containing 10% fetal bovine serum and 1% penicillin/streptomycin (Gibco, Waltham, MA, USA). Cell samples were incubated at 37°C and 5% CO_2_, with medium changed every three days.

### Clinical sample data statistics

A total of 150 clinical samples (124 ovarian cancer samples and 56 ovarian samples) were obtained in our research. All cases who offered clinical samples involved in this experiment have signed the Informed Consent Statement at the beginning of this research. The average age of patients in the ovarian cancer group was 46.19 ± 7.85 years old, with 19 cases in FIGO I stage, 38 cases in stage II, 42 cases in stage III, and 25 cases in stage IV. In addition, 56 patients with normal ovarian tissue diagnosed by myomectomy in our hospital in the same period were selected as the control group. The average age of patients in the control group was 47.42 ± 9.33 years old. This suggested that there was no significant difference in age distribution between these two groups (*p* > 0.05).

### Cell transfection

To build ovarian cancer cell models with different RNA/protein expression (CACNA1G-AS1, IGF2BP1 and FTH1), we cloned sgRNA into the lentiCRISPR v2 vector and mixed it with the packaging plasmids psPAX2 and PMD2.G. After that, we selected ovarian cancer cells in good condition and inoculated them into 6-well plates at 4 × 10^5^ cells/well. When the cell density reached 70%–80%, we placed the cells into 1.5 ml serum-free medium with 500 ml solution containing the transfection vector Lipofectamine 3000. The cell supernatant was replaced with complete medium after 4–6 h, and then we cotransfected the plasmid into SKOV3 and A2780 cells to produce lentivirus particles. After 48 h of transfection, we collected these lentiviral particles and transfected them into SKOV3 and A2780 cells.

### RT‒PCR

Ovarian cancer cells were collected and inoculated into 6-well plates at a density of 5.0 × 10^5^ cells/well. Total RNA was extracted by TRIzol, and all samples were reverse transcribed into cDNA. RNA samples were tested via PCR with GAPDH set as an internal reference, and the relative RNA expression was measured through the 2−∆∆^Cq^ method. Each group was tested independently and repeated three times. The primer sequences were as follows: CACNA1G-AS1 (Forward: 5′-TGTGCTTCACCATGCTCCAT-3′, Reverse: 5′-ATTAGTGCTCCGGCCAACAA-3′); IGFBP1 (Forward: 5′-TTGGGACGCCATCAGTACCTA-3′, Reverse: 5′-TTGGCTAAACTCTCTACGAC TCT-3′); FTH1 (Forward: 5′-TGAAGCTGCAGAACCAACGAGG-3′, Reverse: 5′-GCACACTCCATTGCATTCAGCC-3′). All these primers were provided by GenePharma (Shanghai, China).

### Subcellular localization analysis

We detected the subcellular distribution of CACNA1G-AS1 by lnc-Locator. The cytoplasm and nucleus were isolated using the Cytosolic-Nuclear RNA Purification Kit. Then, the levels of CACNA1G-AS1, GAPDH and U6 were measured through PCR (GAPDH and U6 were used as cytoplasmic and nuclear references, respectively).

### Western blot analysis

We selected a proper number of SKOV3 and A2780 cells and inoculated them into 6-well plates. Then, we lysed these cell samples on ice with RIPA protein lysate and the protease inhibitor PMSF. Next, we proceeded with electrophoresis, assembled a transfer system, and electrotransferred the proteins to membranes; we added the primary antibody (1:1000) and incubated the samples at 4°C overnight. The secondary antibody (1:5000) labeled with horseradish peroxidase and ECL were added in subsequent steps with incubation in the dark at room temperature for 1 h. Finally, we chose ImageJ software to analyze the grayscale values of the protein bands and used GAPDH as an internal reference.

### Colocalization analysis

We selected SKOV3 cells and inoculated them into 6-well plates at a density of 1 × 10^6^/well. Then, we placed these cell samples in 37°C and 5% CO_2_ conditions for incubation. When the cell density reached 70%, we added PFA for fixation (30 min) and 0.5% Triton X-100 for 20 min at room temperature. Goat serum was added to the cell samples and sealed at room temperature for 30 min. Then, we discarded the sealing solutions, added an appropriate amount of primary antibody (IGFBP1), and incubated the cells at 4°C overnight. After that, we added corresponding fluorescent probes (CACNA1G-AS1 and FTH1, green) and fluorescent-labeled secondary antibody (IGFBP1, red) and incubated the samples in a dark room for 1 h. Finally, we added DAPI and anti-fluorescence quenching agent and placed the samples under a fluorescence microscope (FM) for observation.

### RNA immunoprecipitation assay

RIP assays were performed using an immunoprecipitation kit according to the instructions. SKOV3 cells in logarithmic growth phase were selected, washed with PBS, added to RIPA lysis buffer, and lysed on ice for 5 min, after which the cell supernatant was extracted. Magnetic beads coated with AGO2 and IgG were incubated overnight at 4°C. We obtained the protein-RNA complexes once specific proteins were captured, and all cell samples were digested with proteinase K to extract their RNA molecules. Finally, we isolated RNA samples and performed PCR to determine the expression of target genes.

### Intracellular Fe^2+^ detection

We added 200 μl of detection reagent buffer to the samples and placed them under room conditions for 10 min to fully lyse the cells. After that, all samples were centrifuged for 10 min at 4°C and 12000 r/min to obtain 150 μl supernatant, and then 5 μl Fe^2+^ reducing agent was added to each sample and incubated at 37°C for 30 min. Finally, we added 100 μl Fe^2+^ probe, incubated for 1 h and detected the sample absorbance using a colorimetric microplate reader (562 nm wavelength). Following the instructions, we calculated the intracellular Fe^2+^ content according to the standard curve and sample protein concentration.

### Intracellular ROS assay

All cell samples were seeded in 6-well plates and treated for 24 h according to the experimental groups, and the cell precipitate was collected. Then, we diluted the DCFH-DA fluorescent probe (2 μmol/L) or BODIPY™ 581/591 C11 fluorescent probe (7 μmol/L) with serum-free medium. The ovarian cancer cells were resuspended by adding 0.5 ml probe into each well and incubated at 37°C for 30 min. All cell samples were washed twice after shifting with the culture medium, and 150 μl serum-free medium was added to each sample for resuspension. Finally, the fluorescence intensity was tested by flow cytometry.

### Proliferation assay

In the colony formation assay, all cells were inoculated into 6-well plates and cultured with 10% FBS at 37°C and 50% CO_2_. These samples were stained with 0.1% crystal violet 10 days after incubation, and the newborn cells were counted manually. In the EdU assay, the transfected cells were cultured in 96-well plates and treated with 20 μM EdU. After incubation at 37°C and 5% CO_2_ for 2 h, all cells were fixed with 4% PA for 30 min and incubated with 0.5% Triton X-100 in PBS for 20 min. The proliferation rate was calculated according to the instructions, and the cells in each field of view were counted and analyzed using ImageJ.

### Cell migration assay

In this section, ovarian cancer cells were inoculated in 6-well plates for 72 h, supplemented with serum-free DMEM and incubated for 12 h. All cells were washed with PBS 3 times before scratching in the central region with 200 µl pipettor tips. Finally, all cell samples were observed under room conditions (0, 24 h).

### Mitochondrial membrane potential (MMP) assay

Cells were inoculated into 6-well plates at 3 × 10^5^ cells/well and treated for 24 h according to the experimental groups. Then, MMP was detected with JC-1 probes according to the instructions. After staining, all cell samples were analyzed by flow cytometry. The excitation wavelength of JC-1 was set as 488 nm, while the approximate emission wavelengths of the monomeric and J-aggregate forms were set as 529/590 nm, respectively.

### Ultrastructure observation (TEM)

SKOV3 and A2780 cells were inoculated into 6-well plates and treated for 24 h. The cells were digested by trypsin and fixed in 0.1 M phosphate buffer (pH = 7.4) supplemented with 2% PA and 2% glutaraldehyde, after which we fixed these samples with 1% osmium tetroxide for 2 h. The cells were dehydrated in a gradient with different concentrations of ethanol, after which the cells were coincubated with LR white resin (Sigma) for 1 h (twice) and embedded into this resin. The cured blocks were cut into 60 nm diameter sections and stained with uranyl acetate and lead citrate. All cell samples were observed under a TEM, and ultrastructure images were captured. We randomly selected fields of view and observed the morphology of the cytoplasm, nuclear membrane and distribution of nuclear chromatin in the outer nuclear layer to average the results. Digital images were acquired by the AMT imaging system provided by Nanjing Medical University.

### Xenograft study

Ten BALB/c nude mice (21 days) were randomly divided into two groups (NC group and si-CACNA1G-AS1 group). In the NC group, we performed subcutaneous injection with 0.2 ml SKOV3 cells (2 × 10^7^/ml) on the back of each mouse, while in the si-CACNA1G-AS1 group, we chose 0.2 ml CACNA1G-AS1 knockout SKOV3 cells (2 × 10^7^/ml) for injection. During this process, we set the puncture point 1.0 cm from the injection site. Each mouse was injected once, and the next day was marked as the first day after inoculation. Meanwhile, 1 ml PBS buffer supplemented with ferric ammonium citrate (FAC, with 100 μmol/L Fe^2+^) was injected alongside the tumor borders at 0, 6, 12, 18 and 24 days after subcutaneous inoculation of ovarian cancer cells. We chose 6, 12, 18, 24, 30, 36, 42 and 48 days post tumor cell injection as assessment points, where we measured the long/short diameter of tumors with calipers and then calculated tumor volume according to the formula: length × width^2^/2. All tumor-bearing mice were euthanized by intraperitoneal injection with pentobarbital sodium (150–200 mg/kg) on the 50^th^ day, and then these subcutaneous transplant tumors were removed, weighed and analyzed.

### Statistical methods

We proceeded with data analysis with SPSS 26.0 and GraphPad Prism 8.0, and the measurement data with normal distributions are displayed as X ± S. We used variance analysis to compare means among different groups, and a *t* test was used to compare means between two groups. *p* < 0.05 was set as statistically significant (*p < 0.05, **p < 0.01).

## Results

### FTH1 methylation pathway components were highly expressed in ovarian cancer samples

**Figure 1:**
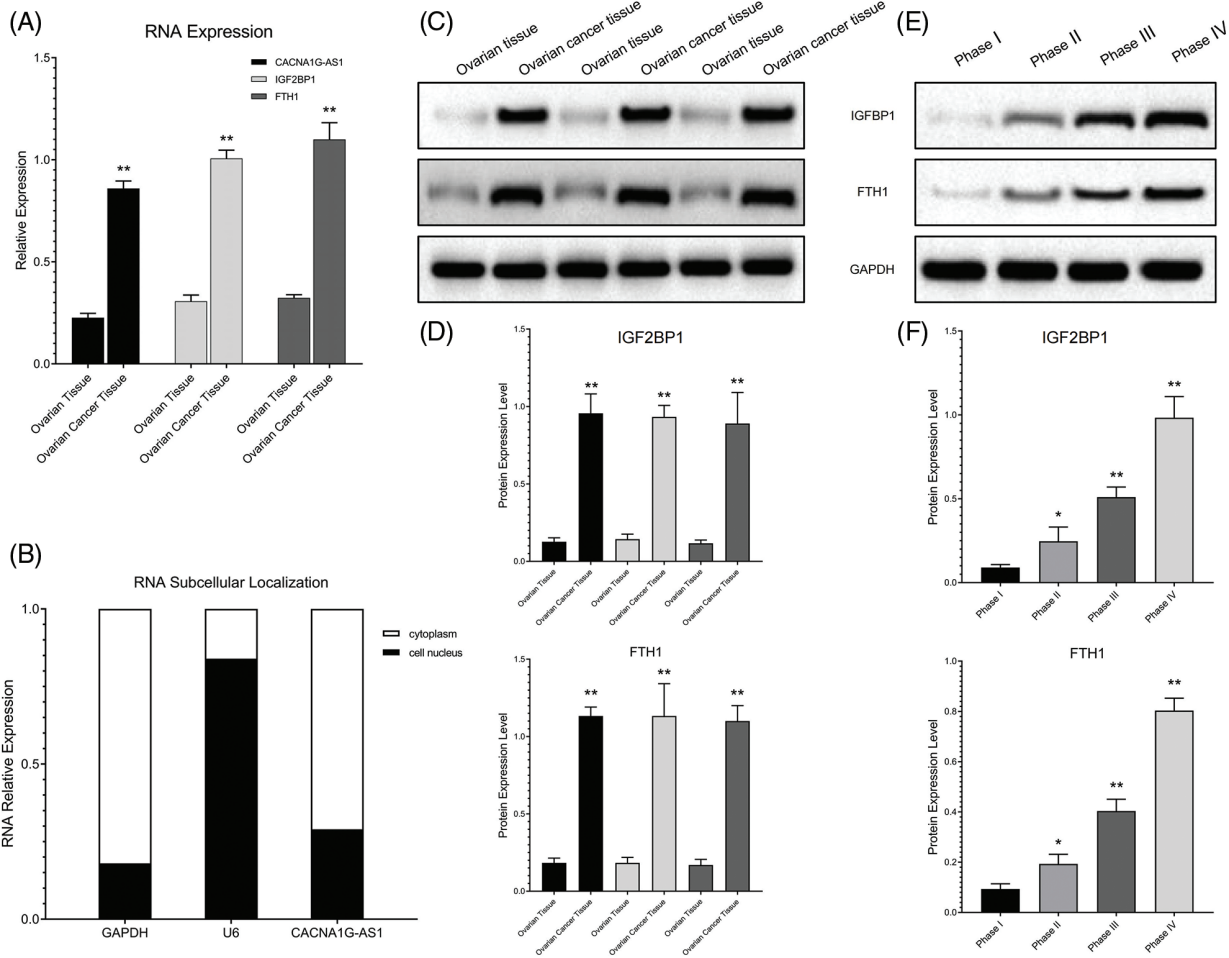
The IGF2BP1 methylation regulatory network showed abnormal expression in ovarian cancer samples. (A) Relative CACNA1G-AS1, IGF2BP1 and FTH1 expression was measured by qRT‒PCR. (B) Subcellular distribution of CACNA1G-AS1 (U6 nucleus internal reference and GAPDH as cytoplasm internal reference). (C and D) Detection of IGF2BP1 and FTH1 protein expression in ovarian tissue and ovarian cancer tissue. (E and F) Detection of IGF2BP1 and FTH1 protein expression in ovarian cancer samples at different stages (FIGO I–IV) (*p < 0.05, **p < 0.01).

We detected the expression of signaling pathway components in cell lines and clinical samples (FIGO I–IV) through PCR and Western blotting (WB) assays. The results confirmed that compared with ovarian cells and tissues, CACNA1G-AS1, IGF2BP1 and FTH1 expression levels were obviously increased in ovarian cancer samples (Figs. 1A, 1C, 1D). The expression of IGF2BP1 and FTH1 was strongly linked to ovarian cancer grade malignancy (Figs. 1E and 1F). Subcellular localization analysis revealed that the expression of GAPDH in the cytoplasm and U6 expression in the nucleus reached nearly 80%, thus confirming that the cytoplasm and nucleus of ovarian cancer cells had been successfully separated. Meanwhile, the expression of CACNA1G-AS1 in the cytoplasm was over 70%, which confirmed that CACNA1G-AS1 was mainly located in the cytoplasm of ovarian cancer cells (Fig. 1B), indicating that this lncRNA might play its own role by regulating posttranscriptional RNA expression. All these results revealed that the FTH1 m6A methylation regulation pathway (CACNA1G-AS1/IGF2BP1) is closely related to ovarian cancer progression.

### IGF2BP1 targets CACNA1G-AS1 and FTH1

**Figure 2:**
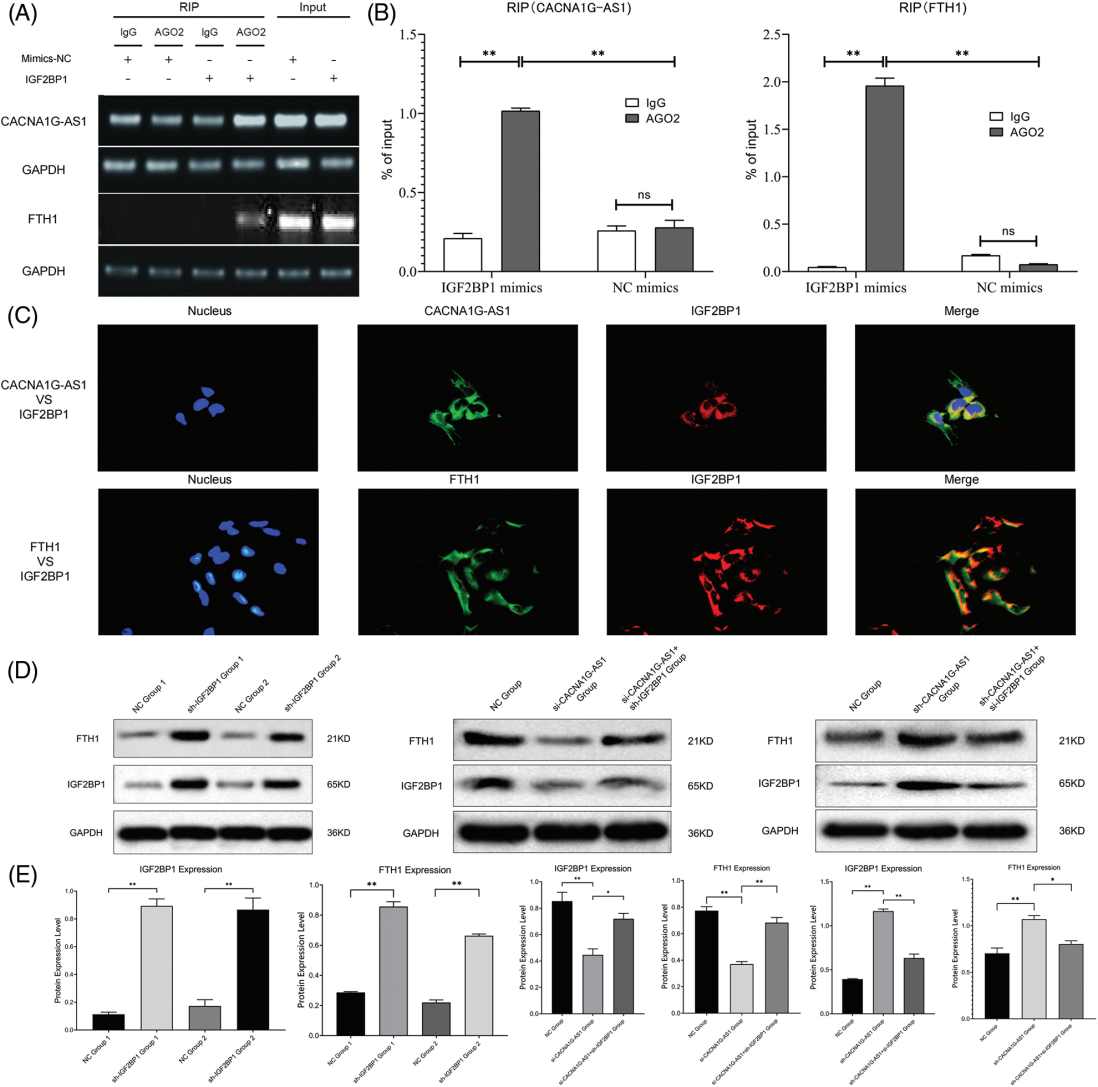
LncRNA CACNA1G-AS1 could bind to IGF2BP1 and then upregulate FTH1 expression. (A and B) RNA immunoprecipitation detection and data statistics (CACNA1G-AS1, FTH1 *vs*. IGF2BP1). (C) Colocalization analysis of pathway factors (CACNA1G-AS1, FTH1 *vs*. IGF2BP1). (D) FTH1 and IGF2BP1 expression was detected through WB after transfection. (E) Statistical analysis of FTH1 and IGF2BP1 expression (*p < 0.05, **p < 0.01).

In this section, we overexpressed IGF2BP1 in ovarian cancer cells and coimmunoprecipitated target RNA with an anti-AGO2 antibody. The results revealed that the contents of CACNA1G-AS1 and FTH1 in IGF2BP1 samples treated with AGO2 showed obvious enrichment compared with those treated with IgG, while the contents of CACNA1G-AS1 and FTH1 in AGO2 samples treated with IGF2BP1 were significantly enriched compared with those in samples treated with mimics-NC. All these results showed that the amount of CACNA1G-AS1 and FTH1 pulled down by the AGO2 antibody significantly increased in ovarian cancer cells transfected with IGF2BP1 (Figs. 2A and 2B). In colocalization analysis, we noticed that CACNA1G-AS1, FTH1 (green) and IGF2BP1 (red) colocalized in the cytoplasm of ovarian cancer cells (Fig. 2C). Based on this, we concluded that IGF2BP1 could bind to CACNA1G-AS1 and FTH1.

### CACNA1G-AS1 promotes FTH1 expression by IGF2BP1-mediated m6A methylation

First, we transfected SKOV3 cells with plasmids containing IGF2BP1 mimics and inhibitors for 48 h and then performed WB assays. The results confirmed that the overexpression of IGF2BP1 could upregulate FTH1, indicating that IGF2BP1 might upregulate downstream FTH1 by promoting m6A methylation. We constructed ovarian cancer cell models with different expression levels of CACNA1G-AS1 and IGF2BP1 and then performed WB to detect FTH1 and IGF2BP1 expression. The results showed that in CACNA1G-AS1 knockdown ovarian cancer cells, FTH1 and IGF2BP1 expression significantly decreased, while IGF2BP1 overexpression reversed this change. In contrast, in CACNA1G-AS1-overexpressing ovarian cancer cells, FTH1 and IGF2BP1 expression obviously increased, while IGF2BP1 downregulation reversed this change (consistent with IGF2BP1, Figs. 2D and 2E). According to these results, we concluded that CACNA1G-AS1 could upregulate FTH1 via IGF2BP1-mediated m6A methylation in ovarian cancer cells.

### CACNA1G-AS1 inhibits ferritinophagy and ferroptosis through the IGF2BP1-FTH1 axis in ovarian cancer cells

In this section, we further investigated whether the CACNA1G-AS1 signaling pathway could regulate ferroptosis in ovarian cancer cells. SKOV3 cells were transfected with plasmids containing RNA mimics and inhibitors (CACNA1G-AS1, IGF2BP1 and FTH1) to construct ovarian cancer cell models with different pathway expression states.

**Figure 3:**
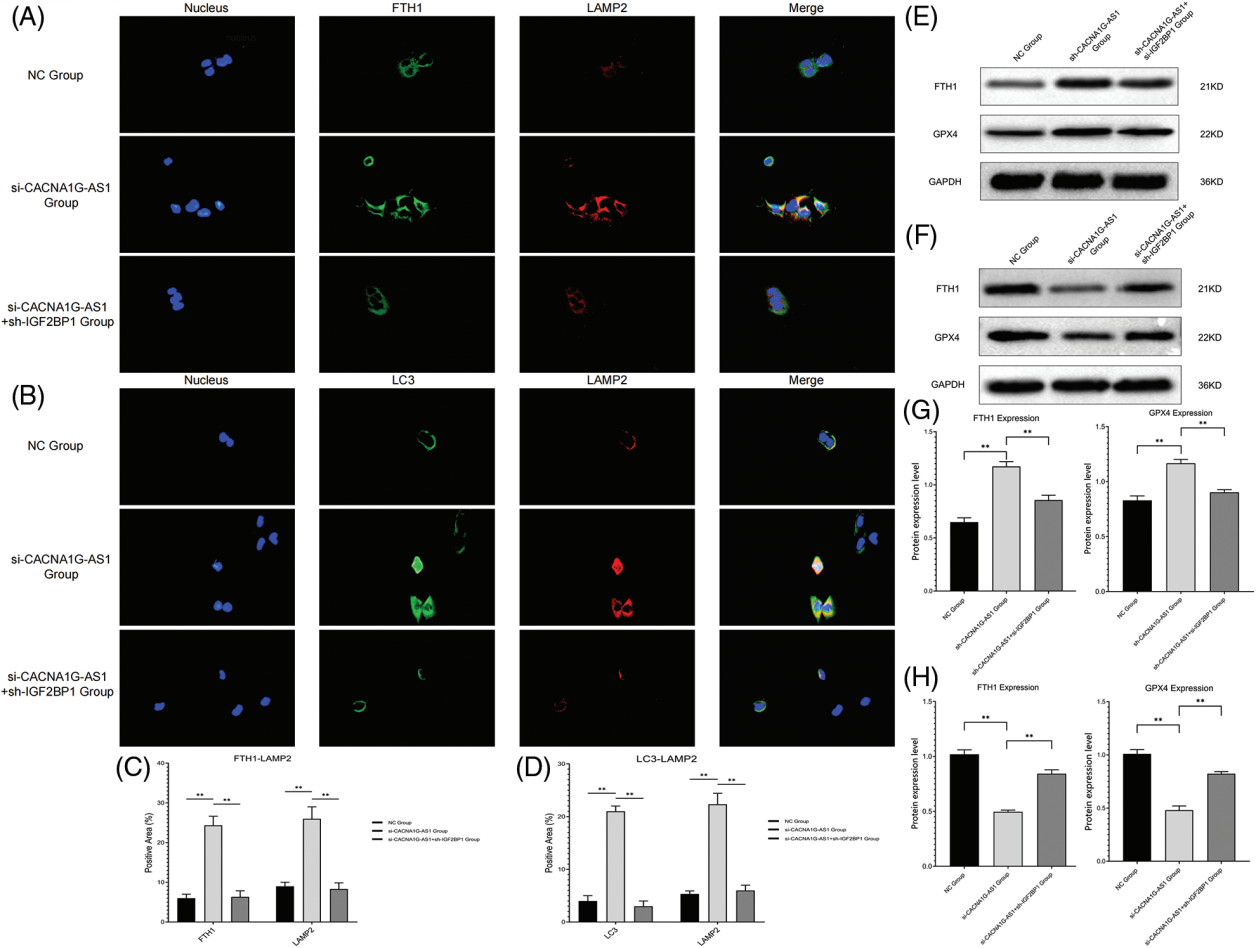
LncRNA CACNA1G-AS1 could inhibit ferritinophagy and ferroptosis by regulating FTH1 expression. (A) Immunofluorescence colocalization analysis of ferritin (green) and lysosome (red) labels. (B) Immunofluorescence colocalization analysis of ferritinophagy (green) and lysosome (red) labels. (C and D) Data analysis of immunofluorescence colocalization. (E, G) WB of FTH1/GPX4 protein expression in the overexpression group and statistical analysis. (F, H) WB of FTH1/GPX4 protein expression in the knockdown group and statistical analysis (**p < 0.01).

First, we detected the colocalization of ferritin and lysosome labels, and the results confirmed that the colocalization of ferritin (FTH1) and lysosome (LAMP2) significantly increased in CACNA1G-AS1 knockdown ovarian cancer cells, while IGF2BP1 upregulation reversed this change. At the same time, we also detected the colocalization of ferritinophagy and lysosome labels. The results revealed that the colocalization of autophagy regulator (LC3) and lysosome (LAMP2) significantly increased in CACNA1G-AS1 knockdown ovarian cancer cells, while IGF2BP1 upregulation reversed this change. According to these results (Figs. 3A–3D), we concluded that CACNA1G-AS1 could inhibit ferritinophagy via IGF2BP1-mediated FTH1 methylation in ovarian cancer cells.

**Figure 4:**
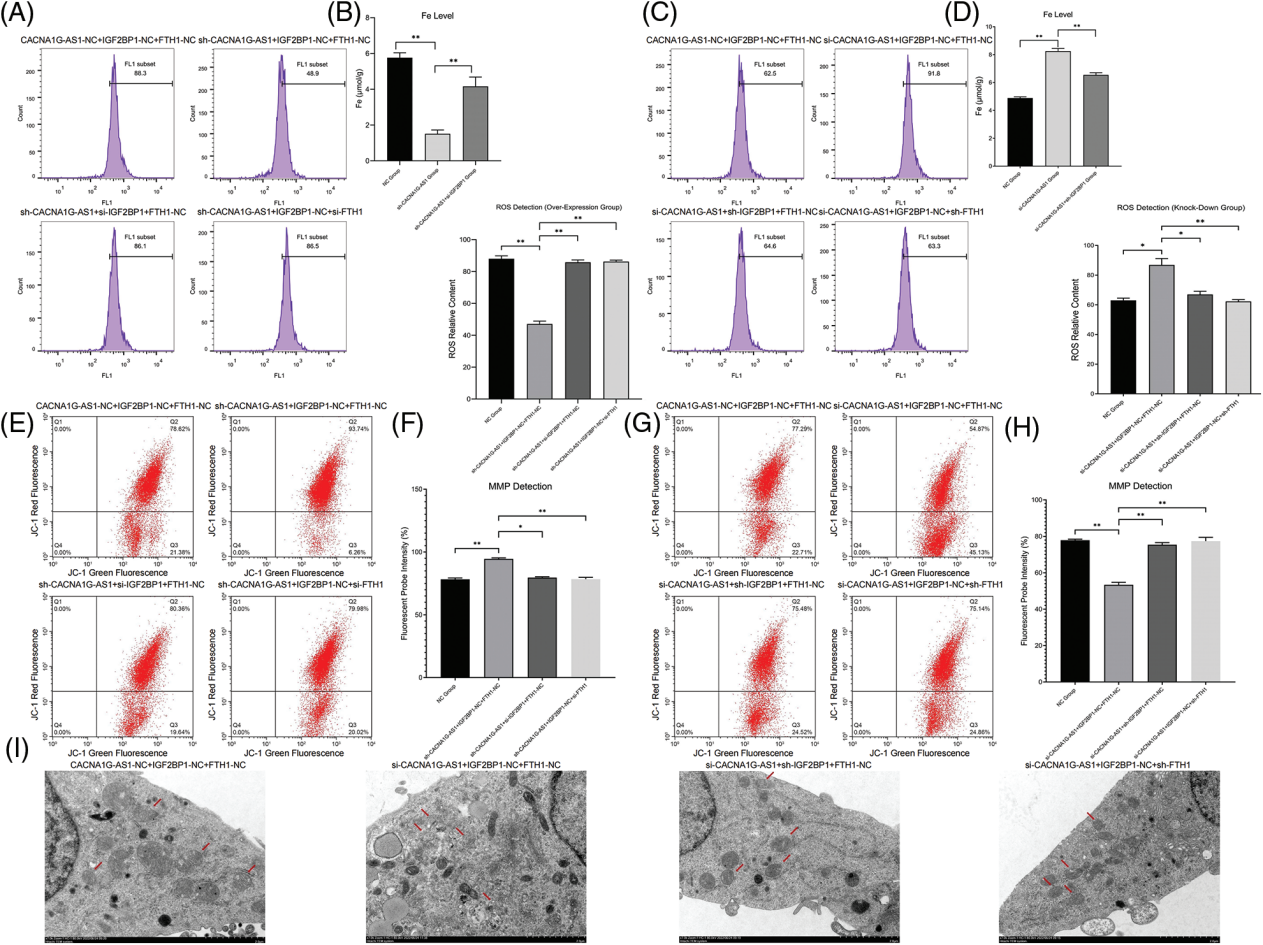
LncRNA CACNA1G-AS1 could reduce ROS content and inhibit mitochondrial autophagy in ovarian cancer cells via the IGF2BP1-FTH1 axis. (A and B) Intracellular ROS and Fe^2+^ detection in the overexpression group and statistical analysis. (C and D) Intracellular ROS and Fe^2+^ detection in the knockdown group and statistical analysis. (E and F) Detection of mitochondrial membrane potential after transfection (overexpression group) and statistical analysis. (G and H) Detection of MMP after transfection (knockdown group) and statistical analysis. (I) Morphological observation of mitochondria by TEM (*p < 0.05, **p < 0.01).

Then, we determined the ferroptosis-related index with Western blotting and a detection kit. The results showed that overexpressed CACNA1G-AS1 could upregulate FTH1 and GPX4, while IGF2BP1 downregulation could reverse this change (Figs. 3E and 3G); moreover, CACNA1G-AS1 knockdown could downregulate FTH1 and GPX4 expression, while IGF2BP1 upregulation could rescue this effect (Figs. 3F and 3H). ROS and Fe^2+^ detection showed that in CACNA1G-AS1-overexpressing ovarian cancer cells, the ROS and Fe^2+^ contents significantly decreased, while these changes could be reversed by IGF2BP1 and FTH1 inhibition. In CACNA1G-AS1 knockdown ovarian cancer cells, the ROS contents significantly increased, and these changes could be reversed by IGF2BP1 and FTH1 overexpression (Figs. 4A and 4B). In contrast, in CACNA1G-AS1 knockdown ovarian cancer cells, the ROS and Fe^2+^ contents significantly increased, while these changes could be reversed by IGF2BP1 and FTH1 upregulation (Figs. 4C and 4D). These experimental results confirmed that CACNA1G-AS1 could inhibit ferroptosis in ovarian cancer cells through the IGF2BP1-FTH1 axis.

### CACNA1G-AS1 inhibits mitophagy through the IGF2BP1-FTH1 axis in ovarian cancer cells

In this section, we used a JC-1 probe to detect the MMP in ovarian cancer cells. The results confirmed that in CACNA1G-AS1-overexpressing ovarian cancer cells, the red fluorescence intensity obviously increased, while these changes could be reversed through IGF2BP1 and FTH1 inhibition (Figs. 4E and 4F). Moreover, in CACNA1G-AS1 knockdown ovarian cancer cells, red fluorescence intensity was obviously reduced, while these changes could be rescued through IGF2BP1 and FTH1 overexpression (Figs. 4G and 4H). Thus, we confirmed that CACNA1G-AS1 could maintain the stability of MMP in ovarian cancer cells through the IGF2BP1-FTH1 axis.

The observation of mitochondrial morphology revealed that in CACNA1G-AS1 knockdown ovarian cancer cells, the mitochondrial volume was obviously reduced, the mitochondrial membrane was ruptured and the cristae vanished, while these changes could be rescued by IGF2BP1 and FTH1 overexpression (Fig. 4I). The above results confirmed that CACNA1G-AS1 could inhibit mitophagy in ovarian cancer cells through the IGF2BP1-FTH1 axis.

### CACNA1G-AS1 promoted the proliferation, migration and in vivo tumorigenesis of ovarian cancer cells through the IGF2BP1-FTH1 axis

**Figure 5:**
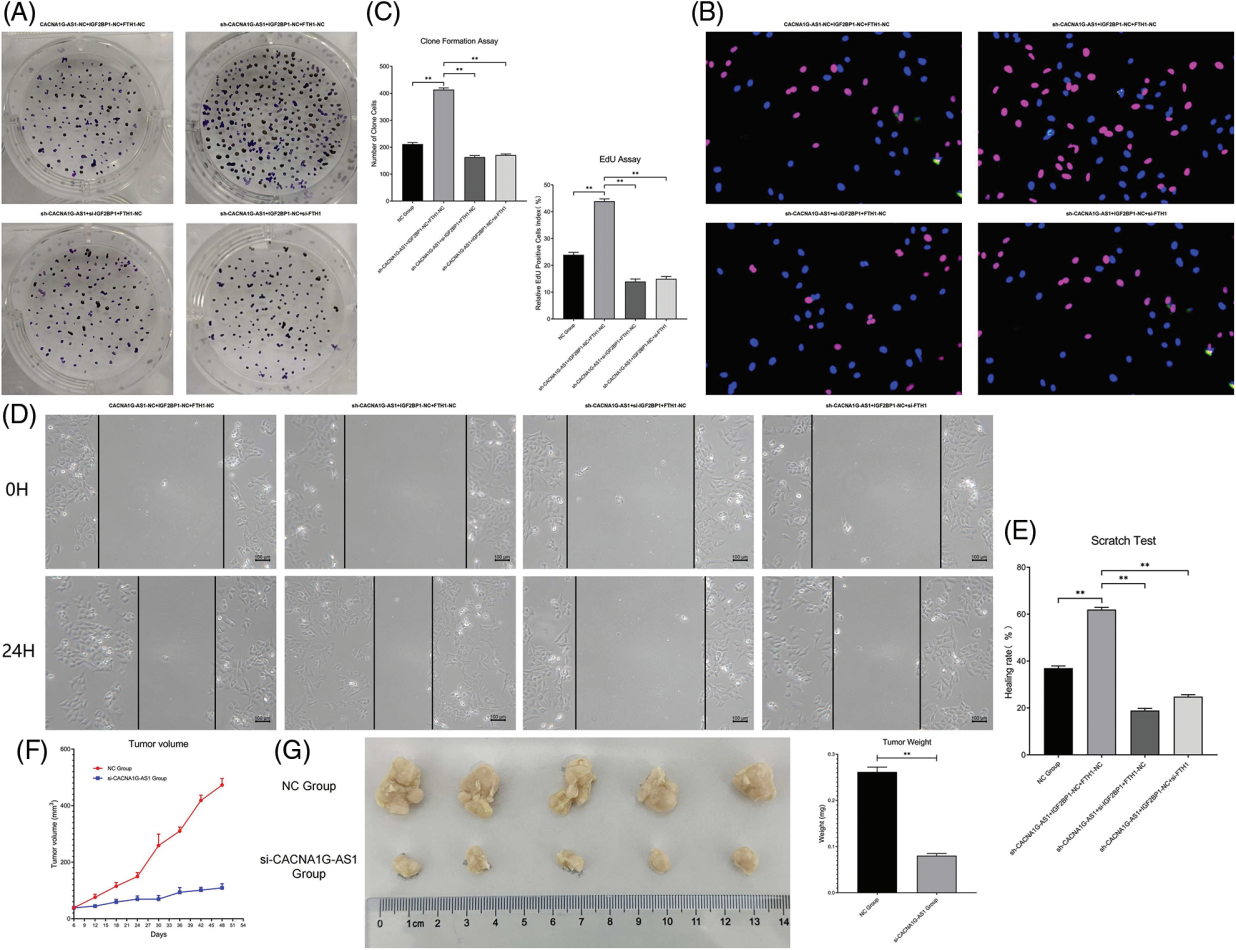
LncRNA CACNA1G-AS1 could promote the malignant phenotype of ovarian cancer cells via the IGF2BP1-FTH1 axis. (A) Colony formation assays of SKOV3 cells after transfection. (B) EdU assays of SKOV3 cells after transfection. (C) Statistical analysis of proliferation detection. (D and E). Scratch test of SKOV3 cells after transfection and statistical analysis. (F) Tumor growth curve *in vivo*. (G and H) Detection of tumor volume/weight under *in vitro* conditions and data analysis (**p < 0.01).

Colony formation assays confirmed that the number of newly generated cells in the CACNA1G-AS1 overexpression group obviously increased, while this number correspondingly decreased after IGF2BP1 and FTH1 inhibition in ovarian cancer cells (*p* < 0.01, Figs. 5A and 5C). EdU assays showed that the proportion of new cells in the CACNA1G-AS1 overexpression group obviously increased (*p* < 0.01), while this proportion correspondingly decreased after IGF2BP1 and FTH1 inhibition in ovarian cancer cells (*p* < 0.01, Figs. 5B and 5C). All these results indicated that CACNA1G-AS1 could promote cell proliferation through the IGF2BP1-FTH1 axis in ovarian cancer cells.

Scratch tests showed that the proportion of transversally migrated cells in CACNA1G-AS1-overexpressing ovarian cancer cells obviously increased (*p* < 0.01), while the proportion of migrated cells correspondingly decreased after IGF2BP1 and FTH1 inhibition (*p* < 0.01, Figs. 5D and 5E). These results confirmed that CACNA1G-AS1 could promote cell migration through the IGF2BP1-FTH1 axis in ovarian cancer cells.

The subcutaneous tumor transplantation experiment showed that compared with that in tumor-bearing mice treated with SKOV3 cells and FAC, the growth rate of tumors *in vivo* significantly slowed (Fig. 5F), while the tumor weight and volume were obviously reduced (*p* < 0.01, Figs. 5G and 5H) in tumor-bearing mice injected with CACNA1G-AS1 knockdown SKOV3 cells and FAC. The above results indicated that CACNA1G-AS1 may decrease the ferroptosis sensitivity of ovarian cancer cells *in vivo*.

## Discussion

At present, it is generally believed that ovarian cancer is the most greatest threat to women’s health, and approximately 150,000 patients die from this disease every year. The current clinical treatment for ovarian cancer patients has not achieved satisfactory results; although remarkable progress has been made in surgery and chemotherapy in recent years, approximately 75% of cases are diagnosed at a late stage due to the absence of typical symptoms. Most scholars believe that the occurrence and progression of ovarian cancer is a multistage process [14–16]. Therefore, further understanding of the molecular mechanism of ovarian cancer occurrence and progression is crucial for the diagnosis and treatment of this disease.

Previous research confirmed that the Fe^2+^ content is strongly linked to various tumors, including lung, liver and ovarian cancers [17]. Basuli built a genetic model related to ovarian cancer initiating cells that indicated the involvement of a reduction in Fe^2+^ efflux pump activity and upregulation of Fe^2+^ transporter expression [18]. Based on this, some scholars considered that targeting Fe^2+^ metabolism in ovarian cancer cells at an early stage might be a potential breakthrough strategy for killing cancer cells.

In 2012, Dixon for the first time defined an Fe-dependent, nonapoptotic cell death mode as “ferroptosis”. He noticed that compared with apoptosis and necrosis, ferroptosis led to decreased mitochondrial cristae loss and mitochondrial membrane rupture, while the cell membrane usually remained intact; therefore, these cells retained a completely spherical appearance. Biochemically, ferroptosis is characterized by increased Fe^2+^ and lipid peroxidation; at present, it is believed that ferroptosis is strongly linked to cysteine metabolism, lipid metabolism and the Fe^2+^ cycle. Cysteine production inhibition and glutathione reduction are the key steps in ferroptosis, and the progression of ferroptosis includes the Fenton reaction, system Xc- and GPX4 [19]. Ferroptosis is considered an Fe^2+^-dependent, lipid peroxide-mediated form of cell death that can be used to selectively remove damaged cells and malignant tumor cells.

Ferritinophagy is a process in which ferritin is specifically recognized and transported to lysosomes for autophagic degradation, thus releasing free Fe^2+^ in normal and tumor cells. Related studies revealed that ferritinophagy could regulate the content of intracellular Fe^2+^ and participate in cell proliferation, differentiation and apoptosis. The disorder of ferritinophagy might lead to the imbalance of iron homeostasis in cells; for example, the lack of ferritinophagy in erythropoiesis could lead to low iron content and induce anemia, while overexpression of ferritinophagy will lead to excessive Fe^2+^ in cells, thus inducing ROS accumulation and ferroptosis. In prostate and breast cancer, FTH1 can regulate immunity and inhibit cell apoptosis through the JNK signaling pathway. FTH1 can also reduce hepatic fibrosis by regulating ferroptosis. In animal experiments, FTH1 knockout in the intestinal tract led to excessive absorption of Fe^2+^ and ultimately promoted cell ferroptosis; catechin might play a neuroprotective role in Fe^2+^ metabolism by upregulating FTH1 expression [20–23].

Previous research revealed that methylation of specific genes is strongly linked to the occurrence, progression, prognosis and chemotherapy resistance of ovarian cancer, and m6A is considered the most frequent RNA modification in eukaryotes. Scholars believe that the abnormal expression of m6A regulatory factors is very important for the malignant phenotype of various tumors. The methylation process of m6A has been proven to be reversible, thus indicating that targeting m6A methylation might be a potential antitumor treatment strategy.

M6A methylation is modified by methyltransferases (writers), demethylases (erasers) and binding proteins (readers). In recent years, studies have revealed that these regulators are significantly differentially expressed in a variety of malignant tumors, and the influence of abnormal m6A methylation mediated by regulators on malignant tumors has been widely investigated [24,25]. Some scholars proved that binding proteins could determine the expression trend of target RNAs, thus regulating downstream molecules, pathways and biological functions.

Relevant research has confirmed that there are three types of m6A binding proteins: the YTH, HNRNP and IGF families. Among them, the IGF2 mRNA binding protein family can recognize m6A-modified mRNA, enhance its stability and promote its translation; in this family, IGF2 mRNA binding protein 1 (IGF2BP1) has the strongest carcinogenic effect. Various studies have confirmed that IGF2BP1 can act as an oncogene in multiple tumors and promote tumor cell proliferation, invasion and chemotherapy resistance. Scholars have proven that IGF2BP1 can upregulate serum response factor (SRF) expression by a m6A-mediated decrease in mRNA decay, thereby enhancing tumor cell proliferation and invasion. Abnormally expressed circ-PTPRA eliminated the enhancement of the malignant cell phenotype mediated by IGF2BP1 in bladder cancer. LIN28B-AS1 targeted binding to IGF2BP1, thus promoting the malignant phenotype in hepatoma cells. Related studies have shown that IGF2BP1 can target the 3′UTR of mRNA and interfere with the regulation of upstream miRNAs. For example, IGF2BP1 could weaken the degradation effect of miRNA on LIN28B, SIRT1 and MAPK6 [26–31]. Li et al. built an ovarian cancer risk score model based on 3 m6A regulatory factors (VIRMA, IGF2BP1 and HNRNPA2B1), thus proving that IGF2BP1 has important value for the prognostic analysis of ovarian cancer patients. The high IGF2BP1 expression group usually has a lower survival rate (5 years and 10 years) and worse clinical prognosis.

Zheng et al. screened 4 lncRNAs that are strongly linked to the methylation and clinical prognosis of ovarian cancer patients (AC010894.3, ACAP2-IT1, CACNA1G-AS1 and UBA6-AS1) through bioinformatics analysis. Target prediction showed that these lncRNAs were bind to METTL5, RBM15, IGF2BP1 and YTHDC1, thus regulating the expression of target mRNAs. Previous studies showed that CACNA1G-AS1 could promote proliferation in hepatoma cells through the miR-2392/C1orf61 axis, and this lncRNA could also regulate the malignant phenotype of non-small cell lung cancer via the HNRNPA2B1 pathway [32–36]. Some scholars have constructed a prognostic grading model of ovarian cancer containing 5 ferroptosis-related factors (ALOX12, ACACA, SLC7A11, FTH1, and CD44) through biological analysis, which showed great value in the prognostic analysis of this disease. Subsequent studies confirmed that FTH1 methylation was closely related to the prognosis of ovarian cancer patients [37–40]. However, until now, there has been no relevant research exploring the upstream methylation regulatory network of FTH1 and its regulatory mechanism on ferroptosis and malignant phenotypes in ovarian cancer.

In this research, we constructed an FTH1 m6A methylation regulatory network in ovarian cancer through signal pathway detection in cell lines and clinical samples. Through pathway expression verification, we confirmed that the FTH1 m6A methylation regulatory pathway (CACNA1G-AS1/IGF2BP1) was obviously increased in ovarian cancer samples, and its expression was closely related to tumor malignancy. Cell model tests revealed that CACNA1G-AS1 could upregulate FTH1 through IGF2BP1-mediated m6A methylation. Ferritinophagy and ferroptosis-related indicator detection showed that CACNA1G-AS1/IGF2BP1-mediated FTH1 m6A methylation could inhibit ferritinophagy and ferroptosis in ovarian cancer cells. Cell function experiments confirmed that CACNA1G-AS1 could inhibit mitophagy and promote malignant phenotypes in ovarian cancer cells through IGF2BP1-mediated FTH1 m6A methylation. *In vivo* studies confirmed that CACNA1G-AS1 knockdown could increase the sensitivity of ovarian cancer cells to ferroptosis induced by Fe^2+^ overload.

## Conclusion

This research confirmed that CACNA1G-AS1 is obviously upregulated in ovarian cancer samples and could upregulate FTH1 through IGF2BP1-mediated m6A methylation. CACNA1G-AS1 inhibits ferroptosis, promotes malignant phenotypes and increases sensitivity to ferroptosis in ovarian cancer cells through IGF2BP1-FTH1-mediated ferritinophagy inhibition. Our research might offer new biomarkers and targets for the clinical evaluation of ovarian cancer patients.

## Future Prospects

The methylation region and expression of FTH1 in ovarian cancer samples have not been clarified, and we plan to address this point by methylation sequencing and detection in the future. In our research, the regulatory mechanism of IGF2BP1-mediated FTH1 methylation in this process and the relationship between ferroptosis and mitophagy in ovarian cancer cells were not fully elucidated, and these topics need to be further explored with subsequent experiments.

## Data Availability

The datasets analyzed in this study are available from the corresponding author on reasonable request.
